# Effects of feeding a vitamin and mineral supplement to cow-calf pairs grazing native range

**DOI:** 10.1093/tas/txad077

**Published:** 2023-07-10

**Authors:** Jennifer L Hurlbert, Friederike Baumgaertner, Kacie L McCarthy, Timothy Long, Cody Wieland, Kevin K Sedivec, Carl R Dahlen

**Affiliations:** Department of Animal Sciences, Center for Nutrition and Pregnancy, North Dakota State University, Fargo, ND 58108, USA; Department of Animal Sciences, Center for Nutrition and Pregnancy, North Dakota State University, Fargo, ND 58108, USA; Central Grasslands Research Extension Center, North Dakota State University, Streeter, ND 58483, USA; Department of Animal Sciences, Center for Nutrition and Pregnancy, North Dakota State University, Fargo, ND 58108, USA; Central Grasslands Research Extension Center, North Dakota State University, Streeter, ND 58483, USA; Central Grasslands Research Extension Center, North Dakota State University, Streeter, ND 58483, USA; Central Grasslands Research Extension Center, North Dakota State University, Streeter, ND 58483, USA; Department of Animal Sciences, Center for Nutrition and Pregnancy, North Dakota State University, Fargo, ND 58108, USA

**Keywords:** cow-calf, grazing, mineral, performance

## Abstract

Our objectives were to evaluate the impacts of providing vitamin and mineral (VTM) supplements to cow-calf pairs during the summer grazing period on cow and calf performance and liver concentrations of minerals. During a two-year period, 727 crossbred cows and their calves (initial cow BW = 601.7 ± 48.1 kg; calf BW = 87.8 ± 5.0 kg; *n* = 381 in year 1, *n* = 346 in year 2) from the Central Grasslands Research Extension Center (Streeter, N.D.) were blocked by parity (**young** [parity 1 to 3], and **old** [parity 4+]) and randomly assigned to pastures at the beginning of the grazing season (16 in year 1 and 14 in year 2). Pastures were assigned to receive a free-choice VTM supplement (**SUPP**) or no VTM supplement (**CON**) from pasture turnout to pasture removal (158 and 156 days in year 1 and 2, respectively). Consecutive day weights were taken from cows and calves at pasture turnout and removal and liver biopsies were collected from a subset of cows at both timepoints and from calves at weaning. Cows were bred via AI 37 to 41 d after pasture turnout and by natural service cleanup bulls for a 70 to 80 d breeding season. Calving and weaning data were collected from the calf conceived and gestated during treatments. Data were analyzed for the effect of VTM treatment (SUPP vs. CON), block of parity, and their interaction using the GLM procedure of SAS with pasture as the experimental unit. Year was considered a random effect in the final analysis. Cow pregnancy success was evaluated using the GLIMMIX procedure in SAS with model terms of VTM treatment, parity, and their interaction with year as a random effect. In year 2, cows in differing days postpartum (DPP) groups at pasture turnout (66.1, 48.8, and 34.5 ± 1.04 DPP for EARLY, MID, and LATE groups, respectively) were selected for liver biopsies with cow as the experimental unit. Cow and calf BW and BW change were not impacted (*P* ≥ 0.20) by VTM access. Pregnancy rate to AI, overall pregnancy rate, gestating calf birth BW and calving distribution were not affected (*P* ≥ 0.11) by treatment. Liver concentrations of Se, Cu, and Co were greater (*P* ≤ 0.002) at pasture removal and weaning for cows and suckling calves that had access to VTM. Cows considered EARLY calving had greater (*P* = 0.05) concentrations of liver Se compared with LATE calving cows. Although VTM supplementation enhanced concentrations of key minerals in the liver of cow-calf pairs, reproductive and growth performance was not affected.

## Introduction

Successful cow-calf herds rely on reproductive efficiency and high-performing progeny to maintain profitability; thus, producers are challenged to seek management practices that may ultimately improve productivity in the herd and thereby enhance their bottom line (Harvey et al., 2021b). Mineral nutrition is an integral component of beef cattle production, impacting numerous aspects of growth and development, reproduction, and immune function (Spears, 2000; Hostetler et al., 2003; Kegley et al., 2016; Arthington and Ranches, 2021). When considering the essential trace elements Se, Cu, Zn, Mn, I, and Co, concentrations of these elements in grazed forages are often observed as being deficient to generally adequate in terms of beef cow requirements (Arthington and Ranches, 2021). Additionally, forage mineral availability on pasture can be extremely dependent on forage species, plant maturity, soil characteristics, climatic conditions, and fertilization practices (Greene, 2000; Arthington and Ranches, 2021).

The summer grazing season in the Northern Great Plains often aligns with rebreeding and early gestation for numerous cow-calf herds. Cattle producers may implement vitamin and mineral supplementation strategies for cow-calf pairs consuming forage-based diets during the summer grazing period when forages may be deficient in trace minerals, which can be impactful during critical periods of production such as uterine involution, rebreeding, early pregnancy, and lactation. Ultimately, maternal nutritional status during critical events in early gestation including maternal recognition of pregnancy, implantation, placentation, and fetal organogenesis have the potential to influence pregnancy success, conceptus growth and development, and potential programming outcomes of the fetus (Hostetler et al., 2003; Funston et al., 2010; Kegley et al., 2016; Dahlen et al., 2021; Menezes et al., 2022).

Our laboratories recently evaluated the impact of providing a vitamin and mineral supplement (VTM) to beef heifers during early gestation and have found that when supplementing VTM to heifers through the first 84 d of gestation, fetuses had increased concentrations of trace minerals in the liver and muscle and altered fetal liver weights, altered fetal liver metabolome, greater concentrations of trace minerals in allantoic fluid, and altered placental gene expression compared with fetuses from non-supplemented cohorts (Diniz et al., 2021; Crouse et al., 2022; McCarthy et al., 2022; Menezes et al., 2022, 2023). Though early gestational impacts of VTM on the gestating female and her offspring have been identified during fetal development, the postnatal investigation of offspring exposed to *in utero* VTM supplementation in a cow-calf production setting during the summer grazing season is underexplored and requires further investigation. Thus, in order to mimic a common production scenario and elucidate the impacts of VTM supplementation on cow-calf herds during the summer grazing season and early gestation, the objectives of the present study include evaluating the impacts of VTM on growth performance and mineral status of the dam and suckling calf throughout the grazing period, reproductive success in the dam, birthweight, calving distribution, and growth of the subsequent calf crop. We hypothesized that VTM supplementation to cow-calf pairs grazing native range pastures would impact cow and suckling calf performance, calving characteristics, the subsequent year’s calf performance, and mineral status of cow-calf pairs.

## Materials and Methods

All experiments and methods were performed following accepted guidelines and regulations. The experimental design, animal management, and tissue collection were approved by the North Dakota State University Institutional Animal Care and Use Committee (A18070).

### Experiment Location

The current study was conducted at the Central Grasslands Research Extension Center in south-central North Dakota during the grazing seasons in 2019 and 2020. The climate in the study area is considered temperate with warm summers and cold winters, and 72% of precipitation occurs between May and September, coinciding with the typical grazing season. The growing season is usually from April to September, with the warmest months of July and August having an average temperature of 20.0 ºC. January is typically the coldest month with an average temperature of -12.2 ºC (NDAWN, 2023). Cow-calf pairs were given access to mixed-grass native range pastures consisting of predominately western wheatgrass (*Pascopyrum smithii* [Rydb.] À. Löve), green needlegrass (*Nassella viridula* [Trin.] Barkworth), and blue grama (*Bouteloua gracilis* [Willd. Ex Kunth] Lag. Ex Griffiths). Numerous other vegetative species in the pasture locations include sedges (*Carex* spp.), prairie junegrass (*koeleria macrantha* [Ledeb.] Schult.), sages (*Artemisia* spp.), and goldenrods (*Solidago* spp.). Species including Kentucky bluegrass (*Poa pratensis* L.) and western snowberry (*Symphoricarpos occidentalis* Hook.) provided a significant contribution to the biodiversity of the pastures (Limb et al., 2018). Previous studies in this experimental location observed decreasing crude protein (CP), neutral detergent fiber (NDF), and acid detergent fiber (ADF) over the course of the grazing season from May to September. Crude protein values of native range forages reported over the grazing season by McCarthy et al. (2021) ranged from 5.82% to 9.08%. Total digestible nutrients (TDN) reported during the summer grazing season by McCarthy et al. (2021) also ranged from 60.23% to 63.9% from May to September. Major minerals including Ca, P, and K in native rangelands have been considered adequate in this experimental location in previous work by Wanchuk et al. (2023), but Mg has been considered inadequate in forages in this area (Wanchuk et al., 2023). Trace minerals including Fe, Mo, Co, and Mn have historically been considered adequate in this experimental location based on NASEM (2016) recommendations, but Cu and Zn have been considered variable and inadequate at times during the grazing season (McCarthy et al., 2021; Wanchuk et al., 2023).

### Animals, Housing, and Diet

During a two-year period, 727 Angus-based primiparous and multiparous cows [initial body weight (BW) = 601.7 ± 48.1 kg] (*n* = 381 in year 1, *n* = 346 in year 2) along with their suckling calves (initial BW = 87.8 ± 5.0 kg; 39 ± 13 d of age) were used to evaluate the influence of providing free-choice vitamin and trace mineral (VTM) supplements during the grazing season on cow-calf herd performance and mineral status. Native range pastures were assigned randomly to one of two treatments: 1) free-choice VTM supplement was available in the pasture (**SUPP**; *n* = 8 pastures in year 1 and 7 pastures in year 2) or 2) no VTM supplement was available in the pasture (**CON**; *n* = 8 pastures in year 1 and 7 pastures in year 2). Pastures were 60.2 ha on average with a range of 43.7 to 103.2 ha. The experiment was designed as a 2 × 2 factorial with main factors of cow parity block (**young** [1st to 3rd parity], and **old** [≥4 parities]), VTM supplement treatment (CON or SUPP), and the respective interaction of block and treatment repeated over 2 years. Within each parity group, cows were ranked by parity number, days postpartum, and BW, then assigned randomly to one of 16 pastures in year 1 and one of 14 pastures in year 2, with cows re-randomized between years 1 and 2 of pasture treatment. The resultant cow parity/treatment combinations were CON/Young (*n* = 4 pastures in year 1; *n* = 3 in year 2), CON/Old (*n* = 4 pastures in year 1; *n* = 4 in year 2), SUPP/Young (*n* = 4 pastures in year 1; *n* = 3 in year 2), and SUPP/Old (*n* = 4 pastures in year 1; *n* = 4 in year 2). Pastures were stocked at a target of 2.27 AUM/ha in 2019 and 2.42 AUM/ha in 2020 averaging 23 pairs per pasture with a range of 16 to 29 pairs assigned to each pasture. Cows remained on their assigned pasture with their suckling calf from pasture turnout on May 18th in both years 1 and 2 and at removal from pasture on October 23rd in year 1 and October 21st in year 2 which also coincided with weaning of the suckling calf. The grazing season for cow-calf pairs in years 1 and 2 lasted 158 and 156 days, respectively. Cows that produced and weaned a calf in both years 1 and 2 were repeated in the experiment, and cows that lost calves between birth and weaning or did not become pregnant were culled from the herd. Primiparous cows were added to the study group in year 2 to account for cows culled from the herd in year 1. Furthermore, treatments were applied to pastures over a two-year period, but evaluation of cow-calf pairs continued through a third additional year of the study.

Prior to the experimental period in each respective year, cows were comingled and had access to VTM while grazing fall crop aftermath for approximately 90 d after pasture removal and received winter feed rations with a VTM supplement incorporated in the total mixed ration (TMR) for 120 d prior to the start of the subsequent grazing season. Diets delivered to all cows during the winter feeding period before project initiation and after summer grazing concluded in each respective year were formulated to meet 100% of requirements specified by the NASEM (2016). Winter diets included prairie hay (10.68% crude protein (CP) on a dry-matter basis (DMB)), corn silage (7.12% CP DMB), and a commercial loose VTM supplement (CHS 12-12+ Mineral; CHS Inc., Sioux Falls, S.D.; Table 1). The VTM supplement used during the winter feeding period was incorporated into the TMR at the common company directed rate of 85 g/hd/d and consisted of a mix of organic and inorganic mineral forms. All cows regardless of previous pasture assignment were managed together and provided a VTM supplement from pasture removal in mid-October to pasture turnout the following year.

**Table 1.** Composition of mineral supplement consumed by cow-calf pairs grazing native range in years 1 and 2 and during the winter feeding period each year; company guaranteed analysis^a,b,c^

	Year 1^a^		Year 2^b^		Winter Feeding^c^	
Item	Min	Max	Min	Max	Min	Max
Minerals						
Ca, %	14.40	17.20	12.00	14.00	12.00	14.00
P, %	8.00	–	6.00	–	12.00	–
NaCl, %	7.70	9.20	17.50	21.00	3.00	4.00
Mg, %	0.60	–	2.75	–	3.00	–
K, %	0.18	–	–	–	–	–
Mn, mg/kg	5,500	–	3,400	–	3,500	–
Co, mg/kg	65	–	38	–	38	–
Cu, mg/kg	2,400	–	3,000	–	2,500	–
I, mg/kg	130	–	300	–	300	–
Se, mg/kg	34	–	36	–	36	–
Zn, mg/kg	7,820	–	9,000	–	7,500	–
Vitamins, IU/kg						
Vitamin A	473,994	–	551,115	–	661,387	–
Vitamin D	117,947	–	55,115	–	66,139	–
Vitamin E	5,880	–	551	–	1,102	–

^a^Stockmen’s Supply Repromune Min YC (Stockmen’s Supply, Inc., West Fargo, N.D.). Ingredients: calcium carbonate, monocalcium phosphate, dicalcium phosphate, salt, magnesium oxide, corn distillers dried grains with solubles, yeast culture, vitamin A acetate, vitamin D_3_ supplement, vitamin E supplement, cobalt carbonate, potassium sulfate, magnesium sulfate, basic copper chloride, copper sulfate, ethylenediamine dihydriodide, manganese sulfate, sodium selenite, zinc sulfate, vegetable and animal fat (preserved with BHT).

^b^CHS 12-6+ Research Mineral (CHS, Inc., Sioux Falls, S.D.). Ingredients: Monocalcium phosphate, dicalcium phosphate, salt processed grain byproducts, calcium carbonate, magnesium oxide, zinc sulfate, manganese sulfate, basic copper chloride, ethylenediamine dihydriodide, cobalt carbonate, sodium selenite, mineral oil, yeast culture, calcium silicate, calcium salts of long chain fatty acids, silicon dioxide, vitamin A supplement, vitamin D_3_ supplement, vitamin E supplement, natural and artificial flavors added.

^c^CHS Ultramin 12-12+ Mineral (CHS, Inc., Sioux Falls, S.D.). Ingredients: Monocalcium phosphate, dicalcium phosphate, processed grain byproducts, calcium carbonate, magnesium oxide, salt, yeast culture, mineral oil, zinc amino acid complex, manganese amino acid complex, copper amino acid complex, zinc sulfate, manganese sulfate, basic copper chloride, ethylenediamine dihydriodide, cobalt carbonate, sodium selenite, natural and artificial flavors added, vitamin A supplement, vitamin D_3_ supplement, vitamin E supplement, calcium silicate, calcium salts of long chain fatty acids, silicon dioxide.

The VTM supplement offered in year 1 was Stockmen’s Supply Repromune Min YC (Stockmen’s Nutrition, West Fargo, N.D.) and in year 2, the supplement offered was CHS 12-6+ Research Mineral (CHS Inc., Sioux Falls, S.D.). Ingredient composition of the VTM supplements and chemical analyses are shown in Table 1. Both supplements used during the summer grazing season have similar formulations and consist of predominately inorganic mineral forms. The VTM supplement on native range was offered in covered mineral feeders placed in a single location near common loafing areas in each pasture assigned to the SUPP treatment. Consumption of the VTM supplement was monitored throughout the grazing season with suggested label intake of 56.7 to 85.0 g/hd/d with the assumption that calves would also be accessing the VTM supplement. Mineral feeders were filled 1 to 2 times per week to ensure that cows and their suckling calves receiving the VTM treatment had access to the targeted amounts of free-choice VTM supplement during the grazing season. Consumption of the VTM supplement was estimated by calculating the sum of total mineral disappearance in each SUPP pasture over the grazing season, then dividing by total cow-calf pairs in the respective pasture and days in the grazing season. Estimated disappearance rate of the VTM supplement was averaged among all SUPP pastures within each year of the study. Loose salt was provided to all cow-calf pairs on CON and SUPP pastures.

### Cow-Calf Performance and Reproductive Measures

Performance of cows and their calves (both suckling and gestating) were evaluated at key times throughout the experimental period, which included measurements at calving, pasture turn out, and pasture removal (weaning). The suckling calf was considered the calf at-side with the dam during years 1 and 2 that had access (or no access) to consuming the mineral supplement with their dam. The gestating calf, however, was the calf that was conceived and gestated during the summer grazing season when treatments were applied and born the following spring. The gestating calf that was conceived and gestated in year 1 was evaluated in year 2, while the calf conceived and gestated in year 2 during the grazing season was evaluated in a third year at birth, pasture turn out, and weaning.

Consecutive two-day cow and suckling calf body weights (BW) were recorded at pasture turnout on May 18th in both years 1 and 2 and at removal from pasture on October 23rd in year 1 and October 21st in year 2 (average calf weaning age: 196 ± 13 d of age). An average of the two-day BW for turn out and removal were used to calculate cow and suckling calf performance on pasture. Gain during the grazing period and average daily gain were calculated for cows and suckling calves. In each year cows were synchronized using a 7-day CO-Synch + CIDR artificial insemination (AI) protocol (Lamb et al., 2010) and bred via AI to multiple sires on June 28th in year 1 and June 25th in year 2. Bulls for natural-service breeding were turned out 5 days after AI and remained with the cow herd for 70 to 80 d, from late June/early July to early September. Pregnancy status was determined via transrectal ultrasonography at least 40 days after bull removal to determine pregnancy rate to AI and overall pregnancy rates.

To determine whether treatment had an impact on the calf conceived and gestated during the grazing season we evaluated subsequent calf birth and growth characteristics. Data collected on the gestating calves included date born, birth BW, pasture turnout BW, and weaning BW. Calving records were evaluated to determine calving distribution, and growth characteristics were recorded via consecutive two-day BW at pasture turnout and weaning.

### Liver Biopsy Collection and Mineral Analysis

Liver biopsies were taken at pasture turnout and removal from a subset of randomly selected cows (*n* = 1 per pasture in year 1 [16 total], and 3 per pasture in year 2 [42 total]). Within one week of weaning, liver samples were collected from a subset of calves selected at random (48 calves in year 1 and 42 calves in year 2). In year 2 cows were selected for biopsy to represent different days postpartum (DPP) groups, with 1 cow in each pasture selected to represent early (EARLY; 66.1 ± 1.04 DPP), middle (MID; 48.8 ± 1.04 DPP), and late-calving (LATE; 34.1 ± 1.04 DPP) cows. Because no treatment had been applied at the time of first biopsy collection these samples were used to evaluate the impact of DPP on mineral status at the time of pasture turn out. Cows and calves were restrained in a squeeze chute to perform the liver biopsy procedure outlined by Engle and Spears (2000) and McCarthy et al. (2021). Briefly, a small area of hair was clipped between the 10th and 12th ribs, and 3 mL Lidocaine Injectable-2% (MWI, Boise, I.D.) administered subcutaneously and into the intercostal muscles at the biopsy site. Placement of the target biopsy site was made on an imaginary line from the tubercoxae to the elbow and in the 11th intercostal space. Following a stab incision, samples of liver were collected using a Tru-Cut biopsy trochar (14 g; Becton Dickinson Co., Franklin Lakes, N.J.), then immediately placed on ashless filter paper to blot dry (Whatman 541 Hardened Ashless filter Papers, GE Healthcare Bio-Sciences, Pittsburg, P.A.) and inserted in tubes designed for trace mineral analysis (potassium ethylenediaminetetraacetate; Becton Dickinson Co., Franklin Lakes, N.J.). Samples were then stored at −20 ºC until further analysis. Samples were analyzed for concentrations of Se, Cu, Zn, Mo, Mn, and Co at the Diagnostic Center for Population and Animal Health at Michigan State University using inductively coupled plasma mass spectrometry.

### Statistical Analysis

Data were analyzed as a mean values for calf birth BW, cow and calf BW at pasture turnout and weaning timepoints, subsequent gain calculations, calving distribution, pregnancy rate to AI and overall pregnancy rate, and liver mineral concentrations for the cow and calf within a pasture were used to represent the pasture in the final data set. Cow and calf performance, liver mineral concentrations, and calving characteristics were analyzed for the effect of VTM treatment (SUPP or CON), parity group (Young or Old), and their respective interaction using the GLM procedure of SAS (SAS 9.4; SAS Institute Inc., Cary, N.C.) with pasture as the experimental unit. In the final analysis, year was considered a random effect in the model. In analyses where the interaction of year and parity were not significant, results were presented as the main effect of VTM treatment. Pregnancy rate to AI and overall pregnancy rate were analyzed as binomial data using the GLIMMIX procedure in SAS with model terms of pasture treatment, parity, and their interaction, with year considered a random effect. For concentrations of liver mineral in relation to DPP in year 2, data were analyzed for the effect of DPP group (EARLY, MID, and LATE) with cow as the experimental unit using the GLM procedure of SAS. Evaluation of mineral status in relation to DPP was conducted before treatments were initiated at pasture turn out; thus, only DPP was considered in the model. Results are reported as least square means using the LSMEANS statement in SAS. Differences were considered significant at *P* ≤ 0.05.

## Results and Discussion

### Mineral Supplement Disappearance on Pasture

Estimated disappearance rate of VTM in native range in the first year of the study indicated that cow-calf pairs on SUPP pastures consumed 105.0 ± 5.3 g/d and in the second year consumed 121.7 ± 5.7 g/d of the VTM supplement provided. Individual animal intakes were not evaluated, but we anticipated that calves would consume free-choice minerals alongside their dams, contributing to the total supplement disappearance during the grazing season. In a previous experiment in our laboratory at the same experimental location, we quantified the mineral intake of cows and their suckling calves using an electronic feeding system and found that cows consumed 63.5% of the total VTM supplement in a pasture, whereas their calves consumed 36.5% of the available VTM supplement (McCarthy et al., 2021). By applying these proportional intakes to the current experiment, we speculate that cows on SUPP pastures may have been responsible for the disappearance of approximately 66.7 g/d in the first year of VTM access and 77.3 g/d in the second year of treatment access; both of which align with the respective manufacturers’ feeding recommendations. Additionally, we speculate that suckling calves with mineral supplement access alongside their dams in the present study likely consumed approximately 38.3 g/d and 44.4 g/d in the first and second year of pasture supplementation, respectively. The VTM intake of suckling calves is likely a critical source of their trace mineral status, as well as an important source of minerals disappearance in pastures. Thus, the potential mineral supplement consumption by suckling calves needs to be considered when developing recommendations for mineral offerings on grazing pastures for cow-calf pairs.

### Impacts on Cow Performance

Cow body weight at pasture turnout and pasture removal (weaning), change in cow BW, pregnancy rates, and subsequent calving distribution were not affected by the interaction of pasture treatment and parity (*P* ≥ 0.55). Cow body weight at pasture turnout, pasture removal, gain on pasture, and pregnancy rates to AI and overall (Table 2) and distribution of subsequent calving (Table 3) were not affected by VTM access on pasture (*P* ≥ 0.22). Overall, performance and pregnancy success were acceptable for cows in both treatment groups compared with benchmarking data and previous research reports (Dahlen and Stoltenow, 2015; Larson and White, 2016; Fountain et al., 2021). Additionally, though BW gain during grazing was not affected (*P* = 0.63), old cows were heavier (*P* < 0.0001) at pasture turnout and pasture removal, and calved later in the subsequent calving season (*P* = 0.007) compared with cows in the young block (data not shown).

**Table 2.** Effect of mineral supplement availability on performance of suckled beef cows for years 1 and 2 of pasture treatments

	Treatment^1^			
Item	CON	SUPP	SE	*P*-value^5^
Turn out BW, kg	597.4	599.9	1.39	0.22
Pasture removal BW, kg	624.6	627.1	3.35	0.61
Cow BW change^2^, kg	26.6	26.2	3.42	0.95
Pregnancy rate to AI^3^, %	42.6	49.8	0.03	0.51
Overall pregnancy rate^4^, %	96.5	95.3	0.01	0.32

^1^Treatments were: CON—cows were grazing pastures with no access to a mineral supplement or SUPP—cows were grazing pastures with access to a mineral supplement.

^2^Cow BW change is the difference in BW (kg) from pasture turnout to pasture removal (weaning).

^3^Pregnancy rate to artificial insemination (AI) determined via transrectal ultrasonography at least 40 days following natural service bull removal. Fetuses detected were aged by a trained technician and classified as conceived to AI or natural service.

^4^Overall pregnancy rates included all detected fetuses via transrectal ultrasonography at least 40 days following natural service bull removal.

^5^Significance considered at *P* ≤ 0.05.

**Table 3.** Effect of mineral supplement availability on performance of suckling and gestating calves; combined averages of years 1 and 2 for the suckling calf and averages of years 2 and 3 for the gestating calf

	Treatment^1^			
Item	CON	SUPP	SE	*P*-value^6^
Suckling calf^2^				
Turn out BW, kg	83.89	82.69	0.836	0.32
Weaning BW, kg	269.02	273.39	2.543	0.24
Calf gain, kg^4^	185.48	190.77	2.851	0.20
Calf ADG, kg	1.18	1.22	0.018	0.20
Gestating calf^3^				
Day of calving^5^	17.66	18.06	0.832	0.74
Birth BW, kg	38.51	39.20	0.294	0.11
Turn out BW, kg	87.59	88.61	0.999	0.47
Weaning BW, kg	251.06	252.74	2.239	0.60
Calf gain, kg	163.48	164.18	1.545	0.75
Calf ADG, kg	1.22	1.22	0.024	0.97

^1^Treatments were: CON—calves were grazing pastures where they (along with their dams) had no access to a mineral supplement or SUPP—calves were grazing pastures where they (along with their dams) had access to a mineral supplement.

^2^The suckling calf was considered the calf at-side with the dam during years 1 and 2 that would have had access (or no access) to the mineral supplement with their dam.

^3^The gestating calf was considered the calf conceived during the grazing season when mineral treatment was applied. The gestating calf was exposed to treatment in utero pasture treatment and born the following spring.

^4^Calf gain (kg) is the total BW gain in the calf from pasture turnout to weaning (pasture removal).

^5^Day of calving refers to the average day of the calving season when calves were born.

^6^Significance considered at *P* ≤ 0.05.

Previous work by our group reported similar BW performance in primiparous cows that consumed high levels of minerals compared with those that consumed low levels of minerals on pasture (McCarthy et al., 2021). Similarly, lack of performance differences for cows receiving a trace mineral supplement on pasture was also described by Arthington and Swenson (2004); Ahola et al. (2004), and Mayland et al. (1980) regardless of trace mineral source or method of feeding. Furthermore, Marques et al. (2016), Muehlenbein et al. (2001) and Jalali et al. (2020) reported similar cow BW responses when supplementing trace minerals to pregnant beef cows and heifers during late gestation. Therefore, VTM supplementation was not impactful for cow growth performance throughout the grazing season in our report, which is corroborated by several previous studies by other laboratories. Furthermore, the effect of parity observed in BW results and calving distribution for cows in the old vs. young block was not unanticipated, as older cows would expectedly have greater BW compared to younger cows, produce heavier calves each year, and likely be bred later in the calving season due to greater lactational demands of raising a heavier calf (Brinks et al., 1962). Research efforts evaluating trace mineral supplementation impacts on cow performance throughout the entire gestation is underdeveloped but is a crucial need for providing insight into the physiological responses of long-term supplementation for the growth and reproductive performance of the cow.

Maternal trace mineral intake including Cu, Mn, Se, and Zn has been shown to influence conceptus growth and development and is also thought to have an effect on embryonic survival and growth (Hidiroglou, 1979; Hambidge et al., 1986; Hurley and Keen, 1987; Crites et al., 2022; Dahlen et al., 2022); thus, the absence of supplemental trace minerals in the maternal diet during the periconceptional period may affect critical events related to conception including maternal recognition of pregnancy occurring as early as 16 days post-breeding (Northey and French, 1980; Thatcher et al., 1994). However, changes to reproductive performance in cows in the current report receiving or not receiving trace mineral supplementation on pasture during the periconceptual period and throughout early gestational timepoints were not observed. Similarly, Marques et al. (2016) and Jalali et al. (2020) also reported comparable responses of pregnancy rate to AI and overall pregnancy rate in cows receiving trace mineral supplementation prior to breeding. Alternatively, Muehlenbein et al. (2001) detected greater 30 d pregnancy rates in cows receiving organic Cu sources compared to non-supplemented cohorts, and DiCostanzo et al. (1986) reported fewer days to first estrus and days to first conception in postpartum cows receiving supplemental Mn compared to non-supplemented cows. In addition, Arthington and Swenson (2004) reported greater pregnancy rates and shorter calving intervals in young cows receiving organic VTM sources compared to inorganic VTM sources, and Stanton et al. (2000) reported enhanced pregnancy rates to AI in cows receiving organic compared to inorganic VTM sources. Although some variation exists for reproductive success in response to VTM supplementation, it is important to reiterate the methods of the current study which evaluated only the impacts of offering a free-choice ­inorganic VTM source (or not) during the grazing season. Our results further indicate that removing VTM supplement from cows for the duration of the grazing season after receiving VTM during a winter-feeding program was insufficient to measurably impact cow weight change and pregnancy ­success. Nonetheless, improving pregnancy success to AI and overall pregnancy rate is fundamental to the biological and economical sustainability of cow-calf enterprises (Diskin and Kenny, 2014). Determining the physiological responses to trace mineral supplementation over a longer portion of the production cycle (i.e., throughout gestation) would provide cow-calf producers with valuable information that could affect herd profitability.

### Impacts on the Suckling Calf

Suckling calf BW at pasture turnout, BW at weaning, BW gain on pasture, and calf average daily gain (ADG) were not affected (*P* ≥ 0.67) by the interaction of pasture treatment and parity of the dam, or by VTM access on pasture (*P* ≥ 0.20; Table 3). However, calves suckling cows in the old block were heavier (*P* ≤ 0.0002) at pasture turnout and weaning and had greater BW gain and ADG during the grazing season than calves in the young block (data not shown).

Existing reports considering impacts of trace mineral supplements on suckling beef calves are limited, especially with experimental parameters similar to the current study utilizing a negative control treatment group. Previous work by our group reported by McCarthy et al. (2021) found similar BW and measurements of gain in calves throughout the grazing season when consuming a low or high level of free-choice mineral via electronic feeders on pasture alongside their dams. We anticipated calves to be consuming the mineral supplement alongside their dams in respective SUPP pastures supported by the feeding behaviors of cow-calf pairs observed by Cockwill et al. (2000) and McCarthy et al. (2021). However, because the growth performance of the suckling calf was unaffected by mineral supplementation from pasture turnout to weaning timepoints, perhaps different physiological growth responses for calves grazing SUPP or CON pastures were not detectable given the time designated in the grazing season. Similarly, Arthington and Swenson (2004) reported comparable responses in calf BW at weaning in response to organic or inorganic trace mineral supplementation offered free-choice or limit-fed to the cow-calf pair grazing winter pasture over a 3-year period. In preconditioned beef calves assigned to receive a trace mineral supplement or a control diet, growth performance traits and ADG were similar regardless of dietary trace mineral treatment (Lippolis et al., 2017). In addition, although parity of the dam was impactful for suckling calf performance and BW gain, these results were not surprising as older cows typically raise a heavier calf compared with young cows (Brinks et al., 1962). Our results conclude that growing beef calves consuming VTM supplements alongside their dams appear to not exhibit differences in growth and performance responses compared to non-supplemented cohorts.

### Impacts on the Gestating Calf

Evaluation of data from the calf that was conceived and in early gestation during the mineral treatment period revealed that subsequent birth BW, pasture turnout BW, weaning BW, BW gain from turn out to weaning, and ADG were not affected by the interaction of VTM access on pasture and parity (*P* ≥ 0.35), or the main effect of VTM access on pasture (*P* ≥ 0.11; Table 3). As expected, block (i.e., cow parity) impacted (*P* ≤ 0.05) performance measures of gestating calf BW at birth, pasture turn out, and at weaning, with calves from older cows having greater BW from birth to weaning, greater BW gain and ADG during the grazing period compared with calves born to cows in the young block (data not shown), which is supported by Brinks et al. (1962). Data from our laboratory clearly show that mineral supplementation from at least 60 d before breeding until 84 d of gestation has impacts on placental gene expression, concentrations of energy substrates in fetal fluids, fetal development, concentrations of minerals in fetal liver, muscle, and fluids, and metabolome of fetal liver (Diniz et al., 2021; Menezes et al., 2021, 2022, 2023; Crouse et al., 2022; McCarthy et al., 2022). Though divergent mineral availability during the grazing season was inclusive of the window from pre-breeding to the first 84 d of gestation as highlighted in previous efforts, no differences in calf characteristics were observed in the current report. Part of the reason may be the design of our experiment that used pasture as an experimental unit. Extreme variability exists in mineral intake among members of a herd with access to common mineral sources (McCarthy et al., 2021, 2023), and experiments reporting impacts on fetal development are often more controlled, with animal as the experimental unit and the assurance that pregnant cattle consume a targeted amount of VTM supplement (Marques et al., 2016; Harvey et al., 2021a; Menezes et al., 2022). The winter diet with the inclusion of a VTM supplement for all cows from pasture removal in the fall to pasture turnout in the spring likely allowed cows from both pasture treatments (SUPP and CON) to have an extended period of VTM storage realimentation prior to the next grazing season. This realimentation period from October to May perhaps offset any performance differences in the offspring that we may have observed if differing VTM access would have continued throughout gestation. Another important consideration is the need to monitor offspring growth, physiological, metabolic, and health parameters beyond weaning as impacts of developmental programming during early fetal development may persist and be more apparent in later life (Barker, 1990; Du et al., 2010; Caton et al., 2019).

### Mineral Status

No treatment × parity interactions (*P* ≥ 0.12) or effects of parity (*P* ≥ 0.10) were present for cow liver concentrations of Se, Cu, Zn, Mo, Mn, or Co; therefore, main effects of VTM treatment are presented in Table 4. By design, concentrations of minerals in the liver of the cow were similar (*P* ≥ 0.18) between CON and SUPP cows at pasture turnout (Table 4). Concentrations of Se, Cu and Co at pasture removal, and change in concentrations of Se, Cu, and Co from turn out to pasture removal were greater (*P* ≤ 0.004) in cows on SUPP pastures than those on CON pastures. Concentrations of Zn, Mo and Mn in cows at the time of pasture removal were not influenced (*P* ≥ 0.23) by pasture VTM treatment. Nonetheless, it is important to reinforce that the study was designed to evaluate natural fluctuations in mineral accumulation in cattle over a grazing season and not necessarily to create mineral deficiencies. Concentrations of Se in the liver for cows on SUPP and CON pastures were classified as adequate (1.25 to 2.5 µg/g DM; Kincaid, 2000) at pasture turn out; however, cows assigned to CON pastures had adequate (1.25 to 2.5 µg/g DM; Kincaid, 2000) Se liver concentrations at weaning while cows grazing SUPP pastures had Se liver concentrations classified as high adequate (> 2.5 µg/g DM; Kincaid, 2000). In addition, Cu liver concentrations for cows in SUPP and CON pastures at pasture turnout and at weaning were classified as adequate (125 to 600 µg/g DM; Kincaid, 2000), as were Zn (25 to 200 µg/g DM; Kincaid, 2000) and Co (0.08 to 0.12 µg/g DM; McNaught, 1948). Manganese concentrations in the liver for cows in SUPP and CON pastures were marginal (7 to 15 µg/g DM; Kincaid, 2000) at both biopsy timepoints of pasture turnout and weaning. Liver concentrations of Fe and Mo were considered adequate for cows grazing SUPP and CON pastures at pasture turnout and at weaning (Kincaid, 2000). A previous study at the same experimental location had similar findings in which cows grazing native range with a high level of mineral intake had greater liver concentrations of Se, Cu, and Co compared with low mineral intake cows, but differences in liver Mn, Mo, and Zn for cows in either treatment were not detected (McCarthy et al., 2021). Based on our previous and current findings, supplementing beef cows and heifers with a trace mineral supplement during the breeding season and into the first trimester of gestation enhanced hepatic Se, Cu, and Co mineral concentrations, which may be influential in increasing Se, Cu, and Co supply to the fetus during the first trimester of pregnancy.

**Table 4.** Effects of mineral supplement availability on liver mineral concentrations in suckled cows grazing native range^1^; combined averages of years 1 and 2 of pasture treatment

			Treatment^2^			
Item^3^	Sample		CON	SUPP	SE	*P*-value^5^
			-------µg/g-------			
Se	Turn out		1.87	1.78	0.066	0.29
	Removal		2.23	2.89	0.153	0.004
		CHG^4^	0.35	1.11	0.166	0.003
Cu	Turn out		206.3	182.7	12.57	0.18
	Removal		185.2	305.4	21.88	0.0004
		CHG	-21.1	122.7	24.46	<0.0001
Zn	Turn out		140.8	141.6	8.42	0.95
	Removal		148.9	172.3	13.83	0.23
		CHG	8.12	30.74	15.167	0.29
Mo	Turn out		3.76	3.82	0.120	0.75
	Removal		4.29	4.20	0.094	0.50
		CHG	0.521	0.379	0.127	0.42
Mn	Turn out		11.12	11.40	0.373	0.58
	Removal		11.13	11.43	0.327	0.52
		CHG	0.013	0.021	0.3395	0.99
Co	Turn out		0.239	0.232	0.0105	0.61
	Removal		0.161	0.300	0.0284	0.002
		CHG	-0.078	0.067	0.0320	0.003

^1^For this analysis, mineral concentration values were averaged between years 1 and 2.

^2^Treatments were: CON—Cows were grazing pastures with no access to a vitamin and mineral supplement or SUPP—Cows were grazing pastures with access to a vitamin and mineral supplement.

^3^Mineral concentrations are reported in µg/g on a dry matter basis. Liver samples were analyzed for concentrations of Se, Cu, Zn, Mo, Mn, and Co via inductively coupled plasma mass spectrometry at the Diagnostic Center for Population and Animal Health at Michigan State University (East Lansing, M.I.).

^4^CHG: Change in concentration, which reflects the concentration at pasture removal minus the value from pasture turn out.

^5^Significance considered at *P* ≤ 0.05.

In the liver of the calf, concentrations of Se, Cu, Zn, Mo, and Co were not affected (*P* ≥ 0.17) by the interaction of treatment and parity, or the main effect of parity (*P* ≥ 0.13). Calves on SUPP pastures with young dams, however, had enhanced concentrations of Mn in their livers (*P* = 0.04; data not shown). Calves on SUPP pastures had greater (*P* ≤ 0.002) concentrations of Se, Cu, and Co in their livers compared with calves grazing non-supplemented (Table 5). Liver concentrations of Zn, Mo, and Mn were not different (*P* ≥ 0.21) in calves assigned to SUPP or CON pastures. Cows and calves were both given access to the free-choice mineral supplement in the pastures assigned to the SUPP treatment, so we suspect that calves consumed VTM alongside their dams (Cockwill et al., 2000; McCarthy et al., 2021). Similarly, Moriel and Arthington (2013) evaluated calves offered creep feed fortified with a trace mineral supplement and reported that supplemented calves had greater liver concentrations of Se, Cu, and Co at weaning compared with calves consuming non-supplemented creep feed on summer pasture. Results from Moriel and Arthington (2013) also corroborate our speculation that suckling calves consuming trace mineral supplements on pasture enhanced concentrations of Se, Cu, and Co in calf liver.

**Table 5.** Effects of mineral supplement availability on liver mineral concentrations in suckling calves grazing native range at weaning; combined averages of years 1 and 2.

	Treatment^1^			
Item, µg/g^2^	CON	SUPP	SE	*P*-value^3^
Se	1.62	1.93	0.063	0.002
Cu	49.3	103.3	6.28	<0.0001
Zn	168.1	171.1	6.35	0.73
Mo	3.45	3.22	0.127	0.21
Mn	8.77	9.17	0.255	0.26
Co	0.113	0.174	0.009	<0.0001

^1^Treatments were: CON—calves were grazing pastures alongside their dams with no access to a vitamin and mineral supplement or SUPP—calves were grazing pastures alongside their dams with access to a vitamin and mineral supplement.

^2^Mineral concentrations are reported in µg/g on a dry matter basis. Liver samples were analyzed for concentrations of Se, Cu, Zn, Mo, Mn, and Co via inductively coupled plasma mass spectrometry at the Diagnostic Center for Population and Animal Health at Michigan State University (East Lansing, M.I.).

^3^Significance considered at *P* ≤ 0.05.

The concentrations of Se in the liver for calves grazing alongside their dams on SUPP and CON pastures were both classified as adequate (1.25 to 2.5 µg/g DM; Kincaid, 2000) at weaning. However, liver Cu concentrations were considered marginal (33 to 125 µg/g DM; Kincaid, 2000) for calves in both pasture treatments. The marginal classification of Cu status in calves at weaning on both pasture treatments is likely due to low levels of Cu availability on pasture (Wanchuk et al., 2023) and milk of their dams (NASEM, 2016). Marginal Cu status in calves was further exacerbated when supplement was not provided on pasture to cow-calf pairs. Concentrations of cobalt concentrations in calves from CON and SUPP pastures were within normal or adequate range at weaning (0.08 to 0.12 µg/g DM; McNaught, 1948). Furthermore, liver Zn, Mo, and Mn were considered within normal or adequate range for calves on both pasture treatments at weaning (Kincaid, 2000). Benefits and subsequent roles of calf mineral status at weaning may perhaps be more apparent in an investigation of post-weaning health and immune function in the transition to high-grain diets in the feedlot. Future research directions with the cow herd used in this study include investigating calf health and performance during the backgrounding and feedlot phase in relation to mineral status at weaning. This evaluation may provide support for the role of VTM on subsequent health, immune function, and calf performance in later postnatal periods.

Our year 2 evaluation of time of calving on concentrations of liver mineral at pasture turnout revealed that EARLY calving cows (mean DPP = 66.1) had greater concentrations of Se (*P* = 0.05) compared with LATE calving cows (mean DPP = 34.5), with the MID group being intermediate (mean DPP = 48.8; Table 6). During late gestation, fetal trace mineral demands increase dramatically (Abdelrahman and Kincaid, 1993; Underwood and Suttle, 1999); thus, maternal mineral stores are shunted to the fetus for normal conceptus growth and establishment of a postnatal mineral reserve (Gooneratne and Christensen, 1989; Kincaid, 2000; Fry et al., 2013). This concept of trace mineral contributions to the fetus has been described extensively with respect to Cu in maternal and fetal tissues, in which fetal liver accumulates greater concentrations of Cu as gestation progresses (Gooneratne and Christensen, 1989). Thus, the rationale of investigating cow mineral status at pasture turnout in relation to DPP provides context to the re-alimentation period, or time period of physiological recovery that cows must undergo between calving and the establishment of a new pregnancy (Short et al., 1990; Joner et al., 2018). The differences in concentrations of Se observed among the DPP groups indicate that perhaps an important part of the repair and recovery process in preparation for pregnancy attainment after parturition is the re-alimentation of body stores of trace minerals. At pasture turnout, LATE calving cows had a shorter post-calving period of physiological recovery before the subsequent breeding season, which likely caused depressed maternal liver concentrations due to a more recent departure from fetal demands for trace minerals during late gestation (Gooneratne and Christensen, 1989). Furthermore, the establishment of a new pregnancy is a low priority of nutrient partitioning in cattle as compared to maintenance demands of the dam (Short et al., 1990). Therefore, in a production setting, increased demands are placed on later calving cows to have a shorter period for uterine involution prior to the breeding season in order to maintain productivity by raising one calf every year (Short et al., 1990; Caton and Hess, 2010). Thus, greater mineral nutritional status of early calving dams at pasture turnout and pre-breeding may be partially responsible for the enhanced pregnancy rates observed as cows progress postpartum (Randel, 1990; Short et al., 1990; Stevenson et al., 2003; Hess et al., 2005; Perry et al., 2016). In addition, these data may be used to make the case for providing high quality mineral supplementation before breeding to facilitate re-alimentation of tissue stores of mineral, especially in cows that calved late.

**Table 6.** Effect of days postpartum on concentrations of mineral in the liver of postpartum beef cows at pasture turnout in year 2 of pasture treatment

	Days postpartum group^1^				
Item, µg/g^2^	EARLY	MID	LATE	SE	*P*-value
Se	1.90^x^	1.77^xy^	1.67^y^	0.064	0.05
Cu	242.4	219.9	180.6	19.31	0.08
Zn	137.3	118.4	106.1	10.59	0.12
Mo	3.78	3.86	3.86	0.133	0.87
Mn	11.83	11.59	11.37	0.377	0.69
Co	0.236	0.232	0.255	0.015	0.56

^1^Days postpartum (DPP): EARLY = 66.1; MID = 48.8; LATE = 34.5 ± 1.04 d. Days postpartum were calculated based on calving date in relation to pasture turn out. In year 2, three cows per pasture were selected for liver biopsy and represented each classification of DPP (EARLY, MID, or LATE) in order to evaluate the impact of DPP on mineral status.

^2^Mineral concentrations are reported in µg/g on a dry matter basis. Liver samples were analyzed for concentrations of Se, Cu, Zn, Mo, Mn, and Co via inductively coupled plasma mass spectrometry at the Diagnostic Center for Population and Animal Health at Michigan State University (East Lansing, M.I.).

^x,y^Means lacking common superscript differ (*P* ≤ 0.05).

## Conclusion

Collectively, supplementing a vitamin and mineral supplement to suckled beef cows and their calves did not enhance cow reproductive performance, growth performance, or postnatal growth performance of suckling calves or calves conceived and gestated during dietary treatment on native range. However, Se, Cu, and Co liver mineral concentrations were enhanced by VTM supplementation for the cow and calf at weaning, which may have post-weaning effects related to health and performance that should be evaluated in future efforts. Evaluation of concentrations of liver mineral at the time of pasture turnout indicates that mineral status is related to days postpartum and may be implicit in the relationship among days postpartum and pregnancy attainment. Understanding the role of VTM supplementation strategies and the potential long-term impacts on herd performance, productivity, and ultimately, profitability in the cow and calf is integral for improving management of cow-calf herds in the Northern Great Plains.
